# Microbiological Methods Used in the Enterics for Global Health *Shigella* Surveillance Study

**DOI:** 10.1093/ofid/ofad576

**Published:** 2024-03-25

**Authors:** Bri’Anna Horne, Henry Badji, Md Taufiqur Rahman Bhuiyan, Lucero Romaina Cachique, Jennifer Cornick, Aneeta Hotwani, Jane Juma, John Benjamin Ochieng, Mahamadou Abdou, Evans Apondi, Hannah E Atlas, Alex O Awuor, Kate S Baker, Bubacarr E Ceesay, Mary Charles, Nigel A Cunliffe, Erika Feutz, Sean R Galagan, Ibrehima Guindo, M Jahangir Hossain, Junaid Iqbal, Fatima Jallow, Noumou Yakhouba Keita, Farhana Khanam, Karen L Kotloff, Victor Maiden, Katia Manzanares Villanueva, Oscar Mito, Md Parvej Mosharraf, Joseph Nkeze, Usman N Ikumapayi, Maribel Paredes Olortegui, Patricia B Pavlinac, Tackeshy Pinedo Vasquez, Firdausi Qadri, Farah Naz Qamar, Sonia Qureshi, Nazia Rahman, Aminata Sangare, Sunil Sen, Pablo Peñataro Yori, Mohammad Tahir Yousafzai, Dilruba Ahmed, Khuzwayo C Jere, Margaret N Kosek, Richard Omore, Jasnehta Permala-Booth, Ousman Secka, Sharon M Tennant

**Affiliations:** Center for Vaccine Development and Global Health, University of Maryland School of Medicine, Baltimore, Maryland, USA; Medical Research Council Unit The Gambia, London School of Hygiene and Tropical Medicine, Fajara, The Gambia; Medical Research Council Unit The Gambia, London School of Hygiene and Tropical Medicine, Fajara, The Gambia; International Centre for Diarrhoeal Disease Research, Bangladesh, Dhaka, Bangladesh; Asociación Benéfica PRISMA, Iquitos, Loreto, Peru; Institute of Infection, Veterinary and Ecological Sciences, University of Liverpool, Liverpool, United Kingdom; Malawi Liverpool Wellcome Research Programme, Blantyre, Malawi; Department of Pediatrics and Child Health, The Aga Khan University, Karachi, Pakistan; Centre pour le Développement des Vaccins du Mali, Bamako, Mali; Center for Global Health Research, Kenya Medical Research Institute, Kisumu, Kenya; Centre pour le Développement des Vaccins du Mali, Bamako, Mali; Center for Global Health Research, Kenya Medical Research Institute, Kisumu, Kenya; Department of Global Health, University of Washington, Seattle, Washington, USA; Center for Global Health Research, Kenya Medical Research Institute, Kisumu, Kenya; Institute of Infection, Veterinary and Ecological Sciences, University of Liverpool, Liverpool, United Kingdom; Department of Genetics, University of Cambridge, Cambridge, United Kingdom; Medical Research Council Unit The Gambia, London School of Hygiene and Tropical Medicine, Fajara, The Gambia; Malawi Liverpool Wellcome Research Programme, Blantyre, Malawi; Institute of Infection, Veterinary and Ecological Sciences, University of Liverpool, Liverpool, United Kingdom; Department of Global Health, University of Washington, Seattle, Washington, USA; Department of Global Health, University of Washington, Seattle, Washington, USA; Centre pour le Développement des Vaccins du Mali, Bamako, Mali; Medical Research Council Unit The Gambia, London School of Hygiene and Tropical Medicine, Fajara, The Gambia; Department of Pediatrics and Child Health, The Aga Khan University, Karachi, Pakistan; Medical Research Council Unit The Gambia, London School of Hygiene and Tropical Medicine, Fajara, The Gambia; Centre pour le Développement des Vaccins du Mali, Bamako, Mali; International Centre for Diarrhoeal Disease Research, Bangladesh, Dhaka, Bangladesh; Center for Vaccine Development and Global Health, University of Maryland School of Medicine, Baltimore, Maryland, USA; Medical Research Council Unit The Gambia, London School of Hygiene and Tropical Medicine, Fajara, The Gambia; Department of Pediatrics, University of Maryland School of Medicine, Baltimore, Maryland, USA; Malawi Liverpool Wellcome Research Programme, Blantyre, Malawi; Asociación Benéfica PRISMA, Iquitos, Loreto, Peru; Center for Global Health Research, Kenya Medical Research Institute, Kisumu, Kenya; International Centre for Diarrhoeal Disease Research, Bangladesh, Dhaka, Bangladesh; Center for Vaccine Development and Global Health, University of Maryland School of Medicine, Baltimore, Maryland, USA; Medical Research Council Unit The Gambia, London School of Hygiene and Tropical Medicine, Fajara, The Gambia; Medical Research Council Unit The Gambia, London School of Hygiene and Tropical Medicine, Fajara, The Gambia; Asociación Benéfica PRISMA, Iquitos, Loreto, Peru; Department of Global Health, University of Washington, Seattle, Washington, USA; Asociación Benéfica PRISMA, Iquitos, Loreto, Peru; International Centre for Diarrhoeal Disease Research, Bangladesh, Dhaka, Bangladesh; Department of Pediatrics and Child Health, The Aga Khan University, Karachi, Pakistan; Department of Pediatrics and Child Health, The Aga Khan University, Karachi, Pakistan; International Centre for Diarrhoeal Disease Research, Bangladesh, Dhaka, Bangladesh; Centre pour le Développement des Vaccins du Mali, Bamako, Mali; Center for Vaccine Development and Global Health, University of Maryland School of Medicine, Baltimore, Maryland, USA; Medical Research Council Unit The Gambia, London School of Hygiene and Tropical Medicine, Fajara, The Gambia; Division of Infectious Diseases and International Health, University of Virginia, Charlottesville, Virginia, USA; Department of Pediatrics and Child Health, The Aga Khan University, Karachi, Pakistan; International Centre for Diarrhoeal Disease Research, Bangladesh, Dhaka, Bangladesh; Institute of Infection, Veterinary and Ecological Sciences, University of Liverpool, Liverpool, United Kingdom; Malawi Liverpool Wellcome Research Programme, Blantyre, Malawi; Department of Medical Laboratory Sciences, School of Life Sciences and Health Professions, Kamuzu University of Health Sciences, Blantyre, Malawi; Division of Infectious Diseases and International Health, University of Virginia, Charlottesville, Virginia, USA; Center for Global Health Research, Kenya Medical Research Institute, Kisumu, Kenya; Center for Vaccine Development and Global Health, University of Maryland School of Medicine, Baltimore, Maryland, USA; Medical Research Council Unit The Gambia, London School of Hygiene and Tropical Medicine, Fajara, The Gambia; Medical Research Council Unit The Gambia, London School of Hygiene and Tropical Medicine, Fajara, The Gambia; Center for Vaccine Development and Global Health, University of Maryland School of Medicine, Baltimore, Maryland, USA; Medical Research Council Unit The Gambia, London School of Hygiene and Tropical Medicine, Fajara, The Gambia

**Keywords:** children, diarrhea, dysentery, microbiology, *Shigella*

## Abstract

**Background:**

*Shigella* is a major cause of diarrhea in young children worldwide. Multiple vaccines targeting *Shigella* are in development, and phase 3 clinical trials are imminent to determine efficacy against shigellosis.

**Methods:**

The Enterics for Global Health (EFGH) *Shigella* surveillance study is designed to determine the incidence of medically attended shigellosis in 6- to 35-month-old children in 7 resource-limited settings. Here, we describe the microbiological methods used to isolate and identify *Shigella*. We developed a standardized laboratory protocol for isolation and identification of *Shigella* by culture. This protocol was implemented across all 7 sites, ensuring consistency and comparability of results. Secondary objectives of the study are to determine the antibiotic resistance profiles of *Shigella*, compare isolation of *Shigella* from rectal swabs versus whole stool, and compare isolation of *Shigella* following transport of rectal swabs in Cary-Blair versus a modified buffered glycerol saline transport medium.

**Conclusions:**

Data generated from EFGH using culture methods described herein can potentially be used for microbiological endpoints in future phase 3 clinical trials to evaluate vaccines against shigellosis and for other clinical and public health studies focused on these organisms.

## Teaser

This article describes the standardization and implementation of optimal microbiological methods for the isolation and identification of *Shigella* in the Enterics for Global Health study. Isolation of *Shigella* from whole stool versus rectal swabs and the use of different transport media are compared.

*Shigella* is a leading cause of diarrhea-associated morbidity and mortality worldwide. The Global Enteric Multicenter Study (GEMS) reported that *Shigella* spp were the second leading cause of moderate-to-severe diarrhea in children aged <5 years and the leading bacterial pathogens in children aged 12–23 months and 24–59 months in Asia and Africa [1]. In 2016, *Shigella* spp were the second leading cause of mortality from diarrhea across all ages, accounting for approximately 212 000 deaths and 64 000 of the globally estimated 446 000 diarrheal deaths in children <5 years of age [2].

Conventional microbiological culture offers several advantages over other *Shigella* identification methods. It ensures bacterial isolation for species identification, antibiotic susceptibility testing, whole genome sequencing, and other laboratory studies. *Shigella* spp are mainly isolated through direct culture of stool using xylose-lysine-deoxycholate (XLD) agar, *Salmonella*-*Shigella* (SS) agar, and MacConkey (MAC) agar. A previous study that isolated *Shigella* from 2160 specimens found XLD to be better for *Shigella* isolation than MAC and SS (107 [5.0%] vs 52 [2.4%] and 97 [4.5%] isolates, respectively) [3]. A separate study found that MAC exceeded SS in isolation (83% vs 40%) of *Shigella dysenteriae* type 1 collected from >12 307 rectal swabs [4].

Using transport media to maintain sample integrity is common and increases *Shigella* isolation rates [3, 5]. Buffered glycerol saline (BGS) and Cary-Blair (CB) are transport media used for the isolation of enteric bacteria. BGS has, however, been shown to outperform CB at recovering *Shigella* from samples transported either at room temperature, refrigerated, or frozen (62.5% vs 12.5%, 87.5% vs 62.5%, and 87.5% vs 62.5%, respectively) [5]. However, the liquid nature of BGS may cause contamination if there are leaks/spills, and the high glycerol concentration may inhibit some *Shigella* spp [6]. Consequently, the use of BGS modified with the addition of agar and reduction of glycerol concentration (mBGS) has been explored and reported to yield increased rates of *Shigella* recovery (46/289 [15.9%]] when compared to BGS alone [29/289 [10.0%]) [6].

Whole stool samples are currently recommended for the identification of *Shigella* from the gastrointestinal tract [7]. However, collection is often not feasible (due to stool not being passed) and handling whole stool can be biohazardous. Rectal swabs offer a more practical sampling approach, but there is a paucity of robust data comparing the recovery rates of *Shigella* from stool and rectal swabs. A previous study reported that rectal swab culture alone resulted in a *Shigella* recovery rate double that observed for stool culture alone [8]. More recently, a retrospective review of 480 paired stool and rectal swabs submitted for enteric culture to 2 hospital laboratories reported similar detection of *Shigella* spp in stool (n = 69 [14.3%]) and rectal swabs (n = 68 [14.2%]) [9, 10]. It should be noted that the sample size in both studies was small. Furthermore, it was not determined whether specific *Shigella* spp or serotypes were preferentially cultured from either of the specimens.

Agglutination with diagnostic antisera is the standard method to serotype *Shigella*. This approach identifies the serotype by slide agglutination with a panel of antisera raised against lipopolysaccharide O-antigen [11]. A benefit of this technique is that it does not require specialized laboratory equipment. However, it is time consuming, prone to error, and laborious. Furthermore, some serotypes lack antisera and results obtained using antisera from different commercial companies vary [12, 13]. Multiple molecular serotyping methods have been proposed to overcome the limitations associated with serotyping by agglutination. The majority are polymerase chain reaction (PCR) based and target the *rfb* gene cluster, which encodes the O-antigen [14–16]; in silico serotyping tools also exist for use on whole genome sequence data from *Shigella* [17].

Shigellosis is generally a self-limiting illness. Antibiotic therapy is recommended for adults and children who present with bloody diarrhea to prevent complications and shorten fecal shedding of the organism [18, 19]. The World Health Organization (WHO) currently recommends ciprofloxacin as first-line treatment and pivmecillinam, ceftriaxone, or azithromycin as second-line treatment for shigellosis [20, 21]. Antibiotic therapy for shigellosis has been complicated by the global emergence of antimicrobial resistance (AMR), specifically in *Shigella sonnei. Shigella sonnei* with reduced susceptibility to ciprofloxacin was first described in Japan in 1993 [21, 22]. Subsequently, ciprofloxacin-resistant *S sonnei* has increasingly been reported throughout Asia and has spread globally [23]. As a result, *S sonnei* is included as a WHO priority pathogen against which new antibiotics are urgently needed [24]. Of recent pressing concern is extensively drug-resistant (XDR) *S sonnei* isolates, which exhibit resistance to the following antibiotics: ampicillin, ciprofloxacin, trimethoprim-sulfamethoxazole, third-generation cephalosporins (including ceftriaxone), and azithromycin. Prior to 2022, XDR *S sonnei* were only sporadically reported in Southeast Asia but have since rapidly emerged internationally while remaining yet to be reported on the African continent [25, 26].

Here, we describe a common protocol to sample, transport, culture, serotype, and perform antibiotic susceptibility testing on *Shigella* used in the Enterics for Global Health (EFGH) *Shigella* surveillance study. EFGH is designed to determine the incidence of *Shigella* in 6- to 35-month-old children with diarrhea who visit health facilities in 7 resource-limited settings. Secondary objectives of EFGH are to determine the antibiotic profiles of *Shigella*, compare isolation of *Shigella* from rectal swabs versus whole stool, and compare isolation of *Shigella* following transport of rectal swabs in CB versus a modified BGS (mBGS) transport medium.

## PROTOCOL DEVELOPMENT

The EFGH investigators agreed that the microbiologic protocols must be standardized across the 7 sites (Bangladesh, The Gambia, Kenya, Malawi, Mali, Pakistan, and Peru). Over the span of a year, structured calls were conducted every 2 weeks to plan the laboratory component of the study. Representatives from each site, as well as representatives from the University of Virginia (for TaqMan Array Card [TAC] expertise), the University of Maryland Baltimore (UMB; for clinical microbiology expertise), and the University of Washington (UW; central coordination team), and other experts as needed formed the EFGH laboratory working group (LWG) and participated in each call. The initial calls were led by UW and 4 co-facilitators (representatives from 3 sites and UMB) and discussed decisions that affected the study design and clinical protocol, standard operating procedures (SOPs), and worksheets to capture the raw data and refinement of case report forms (CRFs). The EFGH LWG developed these SOPs with input from researchers in the EFGH network. All 7 sites contributed their expertise, provided candid and constructive criticism, and created a set of consensus SOPs, worksheets, and CRFs that will be implemented at the sites prior to study recruitment. English-language CRFs and informed consent forms can be found at ClinicalTrials.gov (NCT06047821).

## COLLECTION AND PROCESSING OF WHOLE STOOL SPECIMENS AND RECTAL SWABS

Rectal swabs and a stool sample (if available) will be collected from all enrolled children as described elsewhere [27]. Rectal swabs are preferred over whole stool specimens because (1) samples can be collected immediately, (2) swabs can immediately be placed into transport medium, and (3) rectal swabs may limit potential exposure to severe acute respiratory syndrome coronavirus 2 [28].

Three rectal swabs will be collected using nylon flocked swabs (COPAN diagnostics) and stored as follows: (1) A FLOQSwab will be placed in a dry tube for TAC testing, (2) a FecalSwab will be placed in CB medium, and (3) a FLOQSwab will be placed in mBGS medium. mBGS is a transport medium suitable for *Shigella* and *Escherichia coli*. It contains glycerol (15%), a cryoprotectant that preserves bacterial cells at low temperature. The mBGS medium has sodium chloride and mono- and di-potassium phosphate as buffers, phenol red as a buffer indicator, and agar to make it semi-solid to prevent leakage.

A substudy to compare *Shigella* isolation rates from rectal swabs versus whole stool will be performed in Bangladesh and The Gambia. The stool will be placed in a wide-mouth stool container. FLOQSwabs® and a FecalSwab® will be used to touch the stool targeting bloody, slimy, mucoid, or watery areas and placed into the appropriate tubes or transport medium as described above.

Specimens will be stored and transported within 16 hours of collection at 2°C–8°C. A single-use 2°C–8°C temperature monitor (WarmMark, SpotSee or 3M) will be used during transportation to the laboratory for temperature monitoring to ensure sample integrity. Upon laboratory reception, an accessioning form will be completed and samples eligible for processing will be cultured for *Shigella*. The dry swab will be frozen at −80°C for future TAC testing.

## SHIGELLA ISOLATION AND BIOCHEMICAL IDENTIFICATION

### Culture

**Figure 1.**
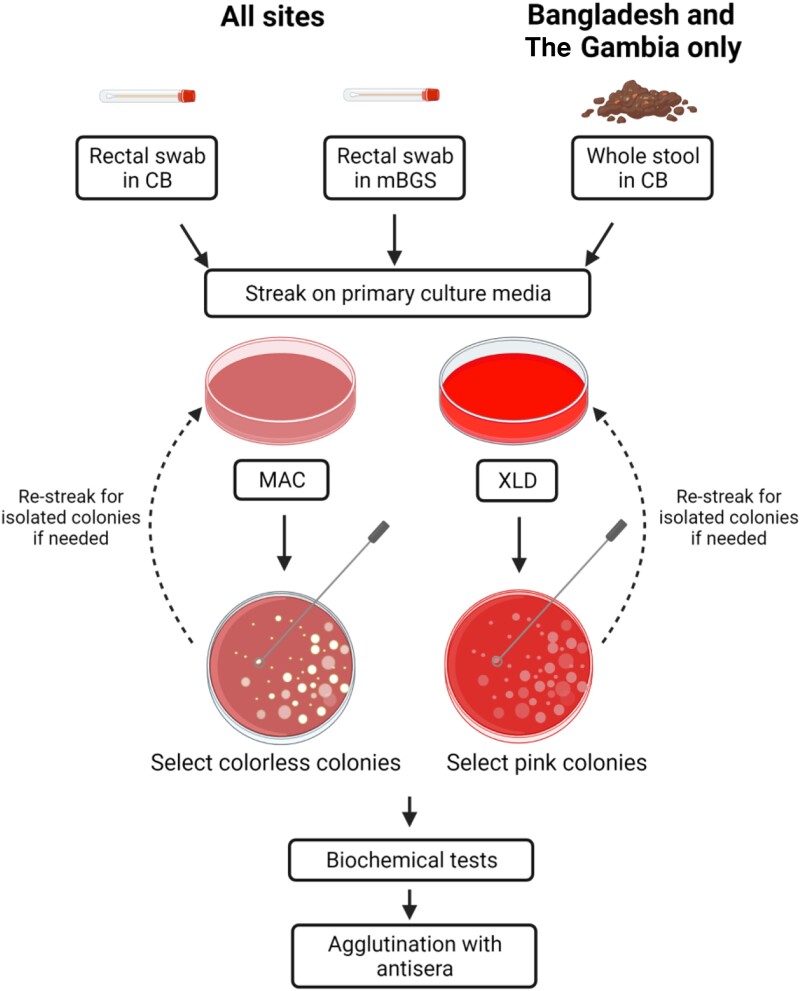
Isolation of *Shigella* spp. from rectal swabs and whole stool.

MAC agar is a differential and low selectivity medium that differentiates Gram-negative bacteria through lactose fermentation and inhibits Gram-positive bacteria and yeasts [29, 30]. Likewise, XLD agar is a differential medium with 3 indicator systems and is a more selective inhibitory medium [31]. Rectal swabs will be used to inoculate the MAC and XLD agar plates and streaked for single colonies (Figure 1). After incubation at 35°C–37°C, up to 10 well-isolated nonlactose fermenter (NLF) colonies (colorless on MAC and pink or red colonies on XLD agar plates) will be subcultured on trypticase soy agar (TSA) plates and screened using a series of biochemical tests. At least 1 representative colony of each suspicious morphotype on each plate, and not to exceed 10 for each participant, will be selected. Colonies will be collected equally from plates streaked from CB versus mBGS where possible. Colonies that are not well isolated (not distinct) will be picked and re-streaked onto fresh plates to ensure that they are pure before subjecting them to biochemical tests.

### Biochemical Tests

Pure NLF colonies from each plate will be inoculated on triple sugar iron (TSI), motility indole ornithine (MIO) or motility indole urea (MIU), and lysine decarboxylase or lysine iron agar tubes for biochemical reactions to identify *Shigella* [32]. TSI characterizes the NLF based on their fermentation of lactose, glucose, and sucrose, and production of gas and hydrogen sulfide (H_2_S). MIO and MIU media are semi-solid and are used to test for motility and indole, urease, or ornithine decarboxylase production by bacteria. The urea in the MIU medium is used to determine the presence of the urease enzyme, which allows for the hydrolyzation of urea to produce ammonia and carbon dioxide. Indole is used to test the ability of the isolates to produce tryptophanase—an enzyme that degrades tryptophan and produces indole, which is a common activity among the Enterobacteriaceae family. Lysine decarboxylase medium is used to test for the ability of the bacteria to utilize the amino acid lysine as a source of carbon and energy for growth. Oxidase test and Gram stain are optional for the sites to exclude other NLFs.

### Expected Results for Shigella

An isolate will be suspected to be *Shigella* if it exhibits the typical biochemical characteristics (Table 1). Colonies will be subsequently serotyped by agglutination with antisera.

**Table 1.** Summary of the Biochemical Tests Used to Identify *Shigella*

Biochemical Test	Activity	*Shigella* Result	Interpretation
TSI medium	Fermentation of lactose and sucrose	Alkaline slant and yellow butt (K/A)	Nonlactose and nonsucrose fermenter
	Production of gas^a^	…	No gas produced
	Production of H_2_S^b^	…	No H_2_S produced
Motility	Swarming in the medium	…	Nonmotile
Urea	Production of urease enzyme^c^	…	No urease production
Indole	Production of tryptophanase enzyme^d^	…	No production of tryptophanase enzyme
Lysine decarboxylase	Production of decarboxylase enzyme^e^	…	No production of decarboxylase enzyme
Oxidase	Production of oxidase enzyme^f^	…	No production of oxidase enzyme

Abbreviations: H_2_S, hydrogen sulfide; K/A, alkaline/acidic; TSI, triple sugar iron.

^a^Gas is observed by appearance of bubbles or cracks in the medium.

^b^H_2_S is observed by black coloration appearing in the butt of the TSI tube.

^c^Urease production is observed by the color change in the medium to pink.

^d^Indole production is observed by a red dye appearing in the surface of the medium upon addition of a few drops of indole reagent.

^e^Carboxylation is observed by change in the medium from purple to yellow in 24 hours of incubation and back to purple after 48 hours of incubation.

^f^Cytochrome oxidase presence is observed by a purple or blue color change in medium or with reagent on filter paper.

## *SHIGELLA* IDENTIFICATION (AGGLUTINATION WITH ANTISERA)

**Figure 2.**
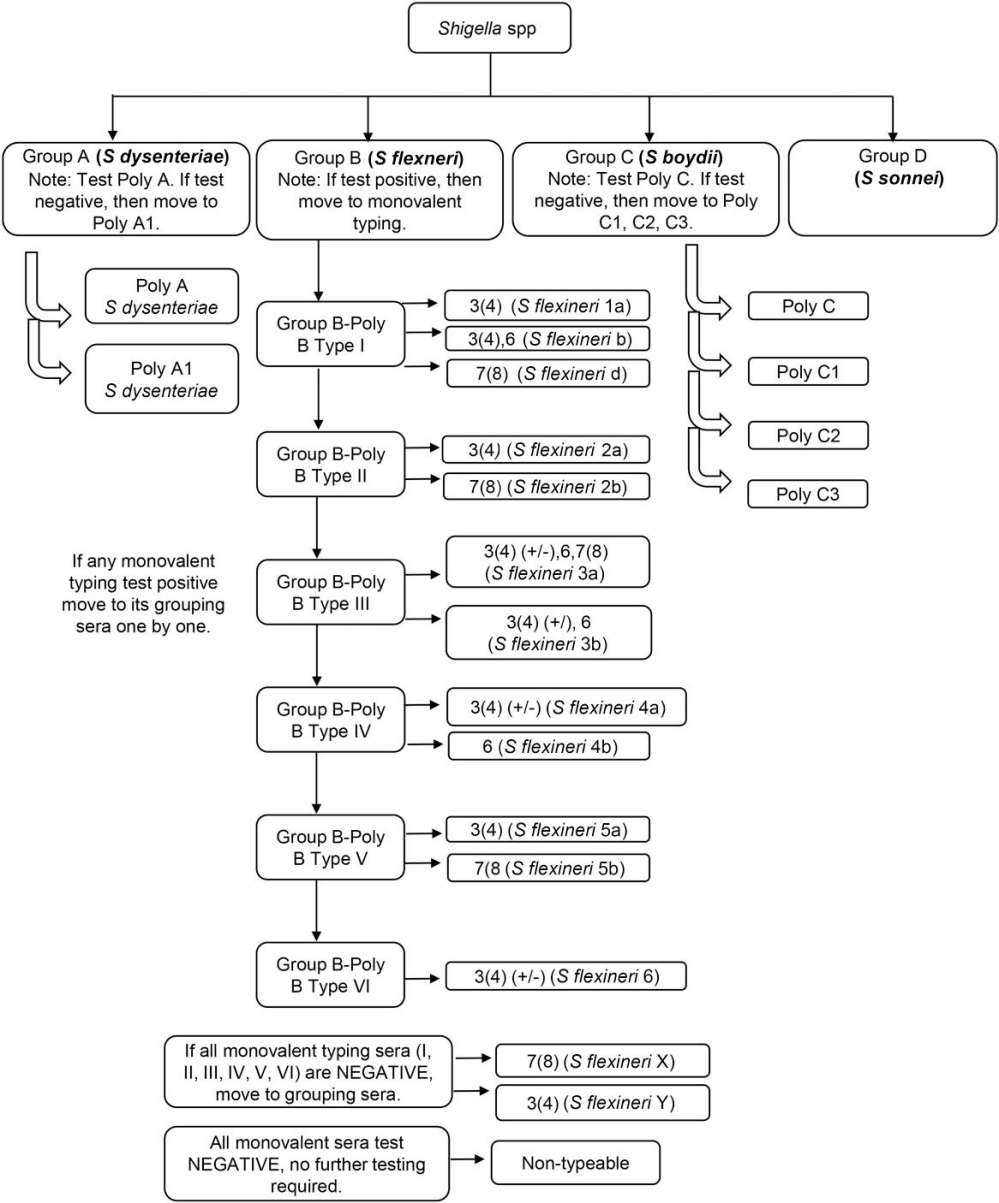
*Shigella* agglutination flowchart using polyvalent and monovalent antisera. Abbreviations: CB, Cary-Blair medium; MAC, MacConkey agar; mBGS, modified buffered glycerol saline; XLD, xylose-lysine-deoxycholate agar.

Fresh and pure cultures of *Shigella* on TSA will be serotyped using polyvalent and monovalent antisera (Figure 2).

1. Polyvalent antisera to identify *S dysenteriae*, *S flexneri*, *S boydii*, and *S sonnei*: For the slide agglutination method, a few glass microscope slides will be sectioned and labeled for each of the antisera (Supplementary Figure 1). A 30-μL drop of physiological saline will be added to the glass slide’s “bacteria only” area as a negative control. Two drops (∼20 μL) of the relevant polyvalent antiserum will be added to their respective sections (Supplementary Figure 1). Two to 5 colonies of *Shigella* will be collected from TSA plates and emulsified. The glass slide will be rocked back and forth for 1 minute and agglutination will be observed. For *S sonnei* (Poly D antisera), slides will be rocked for up to 2 minutes to observe agglutination. Results will be interpreted using Table 2.
2. *Shigella flexneri* serotyping: If the polyvalent agglutination results indicate that the culture is *S flexneri*, agglutination will be performed using monovalent typing antisera (Supplementary Figure 2 and Table 3). Agglutination will be carried out as described for the polyvalent antisera and read after 1 minute. Results will be interpreted using Table 3.
3. Quality control of antisera: All antisera will be verified using positive and negative controls at arrival, every month, and during technical training. Controls consist of a *Shigella* spp–positive control organism and an *E coli*–negative control organism, saline, and antisera in the following combinations: (a) *Shigella* spp plus saline, (b) saline plus antisera, (c) *Shigella* spp plus antisera, (d) *E coli* plus saline, and (e) *E coli* plus antisera (Supplementary Figure 3, Supplementary Tables 1 and 2). Agglutination with the listed antisera will be performed as per the polyvalent antisera and read after 1 minute.

**Table 2.** Interpretation of Agglutination Results Using Polyvalent Antisera

Antigenic Type	Typing Sera								Interpretation
	A	A1	B	C	C1	C2	C3	D	
A	+	−	−	−	−	−	−	−	*Shigella dysenteriae*
A1	−	+	−	−	−	−	−	−	*S dysenteriae*
B	−	−	+	−	−	−	−	−	*Shigella flexneri*
C	−	−	−	+	−	−	−	−	*Shigella boydii*
C1	−	−	−	−	+	−	−	−	*S boydii*
C2	−	−	−	−	−	+	−	−	*S boydii*
C3	−	−	−	−	−	…	+	−	*S boydii*
D	−	−	−	−	−	−	−	+	*Shigella sonnei*

**Table 3.** Interpretation of *Shigella flexneri* Agglutination Results Using Monovalent Antisera

Antigenic Type	Antisera									Interpretation
	Typing Sera						Grouping Sera			
	I	II	III	IV	V	VI	3(4)	6	7(8)	
I:4	+	−	−	−	−	−	+	−	−	*Shigella flexneri* 1a
I:4,6	+	−	−	−	−	−	+	+	−	*S flexneri* 1b
I:7,8	+	−	−	−	−	−	−	−	+	*S flexneri* 1d
II:3,4	−	+	−	−	−	−	+	−	−	*S flexneri* 2a
II:7,8	−	+	−	−	−	−	−	−	+	*S flexneri* 2b
III:(3,4),6,7,8	−	−	+	−	−	−	+/−	+	+	*S flexneri* 3a
III:(3,4),6	−	−	+	−	−	−	+/−	+	−	*S flexneri* 3b
IV:3,4	−	−	−	+	−	−	+/−	−	−	*S flexneri* 4a
IV:6	−	−	−	+	−	−	−	+	−	*S flexneri* 4b
V:3,4	−	−	−	−	+	−	+	−	−	*S flexneri* 5a
V:7,8	−	−	−	−	+	−	−	−	+	*S flexneri* 5b
VI:(4)	−	−	−	−	−	+	+/−	−	−	*S flexneri* 6
−:7,8	−	−	−	−	−	−	−	−	+	*S flexneri* X
−:3,4	−	−	−	−	−	−	+	−	−	*S flexneri* Y

## ANTIMICROBIAL SUSCEPTIBILITY TESTING

Antimicrobial susceptibility testing (AST) will be performed for *Shigella* isolates using the Kirby-Bauer disc diffusion method following the Clinical and Laboratory Standards Institute (CLSI) guidelines [33]. Ampicillin, azithromycin, ceftriaxone, ciprofloxacin, nalidixic acid, mecillinam, and trimethoprim-sulfamethoxazole antibiotic discs (BD BBL Sensi-Disc, Becton Dickinson, Biomaxima, Bio-Rad Laboratories, or Oxoid) will be used. Minimum inhibitory concentration will be determined by Etest for all ambiguous azithromycin results where the zone of inhibition is difficult to measure, particularly for *S sonnei*. Table 4 shows the antibiotics, concentrations, and the CLSI cutoff points that will be used unless they are updated before the study results are to be published. Throughout the study, the results will be reported to the clinical team.

**Table 4.** **Antibiotic Discs, Concentrations, and Clinical and Laboratory Standards Institute** ^a^  **Cut Points**

Antibiotic	Disc Code (µg)	Zone Size Interpretation, mm		
		Sensitive	Intermediate	Resistant
Ampicillin	AMP (10 µg)	≥17	14–16	0–13
Azithromycin	AZM (15 µg)	≥16	11–15	0–10
Ceftriaxone	CRO (30 µg)	≥23	20–22	0–19
Ciprofloxacin	CIP (5 µg)	≥26	22–25	0–21
Nalidixic acid	NA (30 µg)	≥19	14–18	0–13
Mecillinam	MEL (10 µg)	≥15	12–14	0–11
Trimethoprim-sulfamethoxazole	TMP-SMX (1.25/23.75 µg)	≥16	11–15	0–10

^a^Clinical and Laboratory Standards Institute procedures and guidelines, M100, 31st edition.

## QUALITY CONTROL, QUALITY ASSURANCE, AND TRAINING

Quality control (QC) and quality assurance are essential components of any microbiology study, especially when working with multiple laboratories. To ensure that data generated by each laboratory are consistent and reliable, we will implement strong documentation, in-process testing controls, adequate training, regular proficiency testing (PT), and multiple levels of review and feedback. The following procedures will be implemented in this study:

1. Documentation: Detailed SOPs were generated with input from each of the sites in LWG meetings. The SOPs include the use, preparation, and interpretation of media for primary isolation and biochemical tests, agglutination assays for serotyping, and guidelines for AST. Worksheets were developed to track critical steps from each of the SOPs (including details of reagents) and to capture results.
2. Quality controls: In-process QC gives confirmation that the materials, reagents, and media used during the test will provide the expected results. UMB has provided each site with a panel of QC organisms to test the various microbiological assays. The results of these QC tests will be reported in the supporting worksheets for this study.
3. Training: The UW and UMB teams have prepared videos to provide a clear overview of the EFGH work scope. All videos have been made available to all sites. In addition to these recordings, the participating laboratories completed a 5-day on-site didactic and technical training for all microbiological procedures (except for the Bangladesh site staff, who received a 4-hour online training due to travel restrictions from the COVID-19 pandemic). Individual progress for training was captured in 3 parts: observed process, assisted process, and performed process. Each of these progress points allowed the site laboratory staff to gain increased independence on the tested procedures.
4. External quality assurance and proficiency testing: All sites are required to participate in a clinical microbiology external quality assurance (EQA) program of their choosing involving detection and identification of bacteria from blinded samples. Since most of these EQA programs do not include *Shigella*, the UMB team developed a PT program focused on detection and identification of *Shigella*. PT samples will be used to confirm technician skill and comprehension of the procedures. The contents of each PT sample vary and can include pure *Shigella* cultures or mixed cultures. During training, microbiologists at each site were required to complete PT on 2–3 samples, including 1 unblinded and 1–2 blinded samples. Each site microbiologist will be required to test 3 PT samples every 6 months. The results from the PT samples will be reviewed by the UMB team and feedback provided to the sites.
5. Documentation review: Information captured during testing will be recorded in the worksheets in real time and reviewed within 7 days of workup completion by a laboratory supervisor. This information will then be recorded in the relevant CRF. Protocol deviations will be documented and reported to the laboratory supervisor and any critical deviations will be reported to the UMB team. The UMB team will perform a secondary review of 10% of all microbiology documentation (worksheets and CRFs). This review will look for completeness, accuracy, protocol adherence, verification of performer, and supervisor approval.
6. In-person site visits: The UMB team will visit each laboratory approximately every 6 months to review procedures and documents and provide additional training as needed. A laboratory monitoring checklist will be used to record laboratory safety, personnel needs, equipment, materials and inventory, documentation, and procedures (Supplementary Table 3). A report will be provided to each site after each visit and teams will be expected to respond to any findings with a proposed plan for correction, emulating the type of external monitoring that could occur in a vaccine trial.

## CENTRAL PROCUREMENT OF SUPPLIES

To ensure consistency in supplies, the UMB team will provide the following items to the participating laboratories:

1. Antisera: A panel of antisera for serotyping *Shigella* will be provided to all laboratories. The antisera will be shipped to the participating laboratories in temperature-controlled containers and QC tests will be performed upon arrival and monthly thereafter. The antisera will be stored at the recommended temperature, and expiration dates will be monitored.
2. Swabs: The swabs used for sample collection will be shipped to the participating laboratories at room temperature.
3. Temperature monitors: Single-use temperature monitors will be provided to all participating laboratories to ensure that samples are transported at 2°C–8°C.

The participating laboratories are responsible for procuring and maintaining all other laboratory equipment, reagents, and supplies required for the study.

## DISCUSSION

Public health reporting of infectious diseases remains largely dependent on microbiological confirmation, although it is increasingly common to use nucleic acid testing for diagnosis. Specifically, PCR targeting *ipaH* [21, 34, 35], a multicopy gene restricted to *Shigella* spp and enteroinvasive *E coli*, is a sensitive and broadly reactive assay. Microbiologic isolation and characterization methods for *Shigella* have inherent limitations, such as being time consuming and labor intensive and requiring specialized growth media and conditions, and lack sensitivity compared to molecular methods. However, classical microbiological methods have several important advantages to molecular methods. First, since *ipaH* is found in both *Shigella* spp and enteroinvasive *E coli*, there is uncertainty about which bacterium has been detected. In contrast, microbiological methods can discriminate between these organisms, providing more precise estimates of prevalence, which is important for vaccine development and efficacy studies. Second, although the *ipaH* primers/probes have been shown to reliably detect *Shigella* spp, probe sets that are able to differentiate *Shigella* serotypes have not been fully evaluated. It is essential to carefully characterize the serotypes of *Shigella* circulating in high-disease-burden settings to understand shifts in prevalence of individual serotypes and to evaluate potential vaccine efficacy [36]. Furthermore, certain serotypes have been shown to be associated with clinical syndromes or phenotypes and virulence elements [37]. Liu et al have developed real-time PCR assays to detect individual serogroups and serotypes, but these have not yet been tested in a large prospective study across multiple sites [38].

In many countries, clinicians use AST results to guide treatment, which is important given the emergence of strains resistant to fluoroquinolones, azithromycin, ceftriaxone, and other third-generation cephalosporins, and the aggregate data from such testing provide the critical public health function of monitoring bacterial populations for trends in AMR [39]. Nucleic acid testing cannot provide these data because testing is done on whole stool samples and determinants cannot be assigned to individual microbes. The growing emergence of highly resistant *Shigella* strains may affect prioritization for future *Shigella* vaccine rollout, so it is important to characterize the burden of AMR completely and precisely at potential vaccine trial sites. Provision of results to patients and providers has an important role in improving the individual care of trial participants and educating clinicians on the adequacy of standard empiric care.

The preservation of isolates from the EFGH study allows for unequivocal confirmation by whole genome sequencing and could allow for additional studies on microbial ecology, virulence factors, AMR determinants, and epidemiology across regions and globally. Furthermore, these genomic data can be mined to improve treatment and control strategies [40]. Finally, pure *Shigella* cultures can be archived for future studies to develop or evaluate therapeutic and prophylactic strategies to target *Shigella*, to study bacterial pathogenesis, and to investigate phenotypes such as AMR.

In summary, we have described procedures to detect and identify *Shigella* using optimal isolation techniques. We anticipate that our methods will capture an up-to-date assessment of the prevalence and antibiotic resistance profile of *Shigella* at EFGH study sites. The progressive emergence of resistant strains of *Shigella* has intensified the need for the development and evaluation of vaccines to control shigellosis. The techniques described here can be implemented in imminent phase 3 *Shigella* vaccine efficacy studies and can support other surveillance and clinical research studies on these globally important pathogens.

## Supplementary Data

Supplementary materials are available at *Open Forum Infectious Diseases* online. Consisting of data provided by the authors to benefit the reader, the posted materials are not copyedited and are the sole responsibility of the authors, so questions or comments should be addressed to the corresponding author.

## Supplementary Material

ofad576_Supplementary_Data

## Notes

***Acknowledgments.*** We thank Dr Nancy Strockbine, *Escherichia* and *Shigella* Reference Unit, US Centers for Disease Control and Prevention, for input on isolation and identification of *Shigella* spp.

***Author contributions.*** B. H., H. B., M. T. R. B., L. R. C., J. C., A. H., J. J., J. B. O., D. A., K. C. J., M. N. K., R. O., J. P. B., O. S., and S. M. T. actively participated in monthly working group meetings during which the conceptualization and outline was discussed and agreed upon. B. H., H. B., M. T. R. B., L. R. C., J. C., A. H., J. J., and J. B. O. wrote the first draft of the manuscript, and D. A., K. C. J., M. N. K., R. O., J. P. B., O. S., and S. M. T. provided review, scientific input, and editing. E. A., H. E. A., A. O. A., K. B., B. E. C., F. N. Q., M. C., N. A. C., M. A., E. F., S. R. G., I. G., M. J. H., M. T. Y., F. J., J. I., F. K., N. Y. K., F. Q., K. L. K., V. M., O. M., M. P. M., J. N., U. N. I., M. P. O., P. B. P., S. Q., N. R., A. S., S. S., K. M. V., T. P. V., and P. P. Y. reviewed and edited the manuscript. All authors approved the content of the final manuscript.

***Disclaimer.*** The funding organization did not contribute to the design, collection, analysis, or interpretation of the writing in this manuscript. The findings do not necessarily represent the official position of any of the collaborating partners.

***Supplement sponsorship.*** This article appears as part of the supplement “Enterics for Global Health (EFGH) *Shigella* Surveillance Study-Rationale and Methods,” sponsored by the Bill & Melinda Gates Foundation.

***Financial support*.** This project is supported by the Bill & Melinda Gates Foundation (grant numbers INV-016650, INV-031791, INV-036891, INV-036892, INV-028721, and INV-041730) and the National Institutes of Health (award number D43TW010913 to M. N. K. and M. P. O.). The Gambia team's work is also supported by the UK Research and innovation Medical Research Council (program number MC_UU_00031/1, Disease Control and Elimination). Nigel Cunliffe is a National Institute for Health and Care Research (NIHR) Senior Investigator (NIHR203756). Nigel Cunliffe is affiliated to the NIHR Global Health Research Group on Gastrointestinal Infections at the University of Liverpool; and to the NIHR Health Protection Research Unit in Gastrointestinal Infections at the University of Liverpool, a partnership with the UK Health Security Agency in collaboration with the University of Warwick. The views expressed are those of the author(s) and not necessarily those of the NIHR, the Department of Health and Social Care, the UK government or the UK Health Security Agency.
