# Supplementary material for: Microbiological Methods Used in the Enterics for Global Health Shigella Surveillance Study
Source: Open Forum Infect Dis. 2024 Mar 25;11(Suppl 1):S25–33. doi: 10.1093/ofid/ofad576 (PMC10962722; doi:10.1093/ofid/ofad576)
Supplement: ofad576_Supplementary_Data [file ofad576_supplementary_data.docx]

**Supplementary section**

***
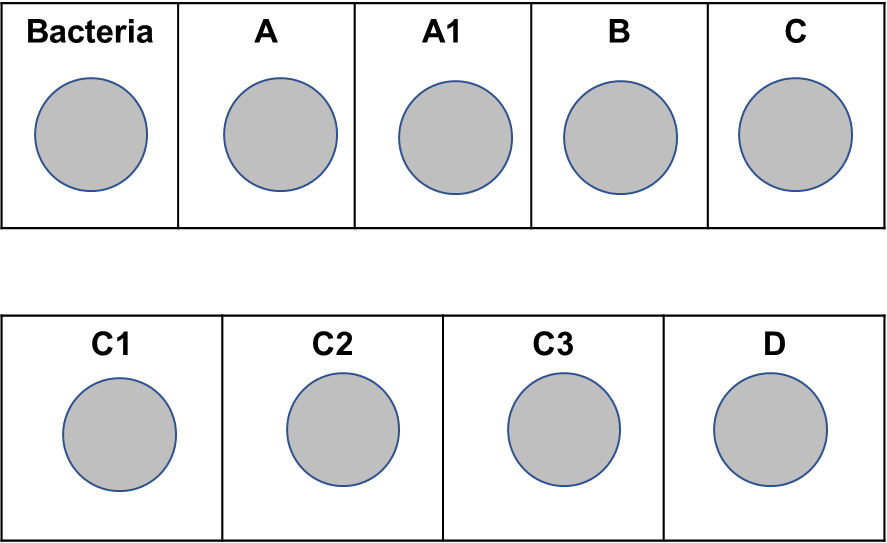
***

**Figure S1.** Layout for agglutination using polyvalent antisera.

**
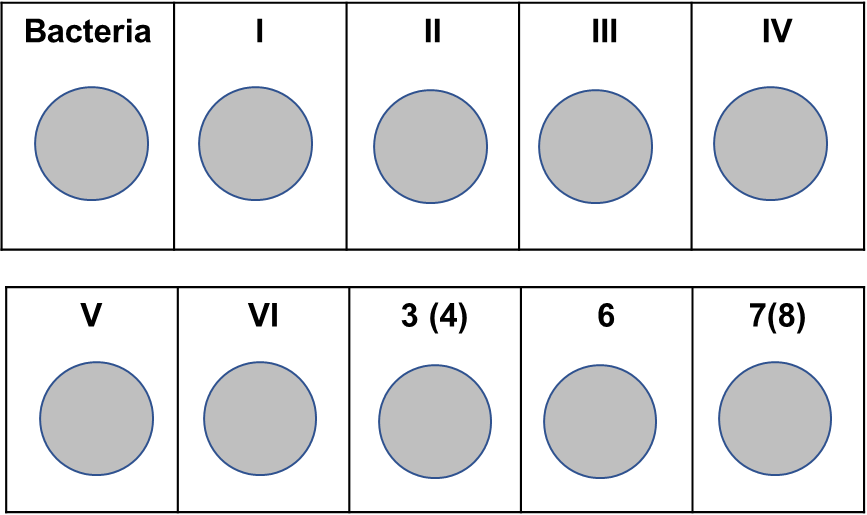
**

**Figure S2.** Layout for agglutination using monovalent antisera.

**
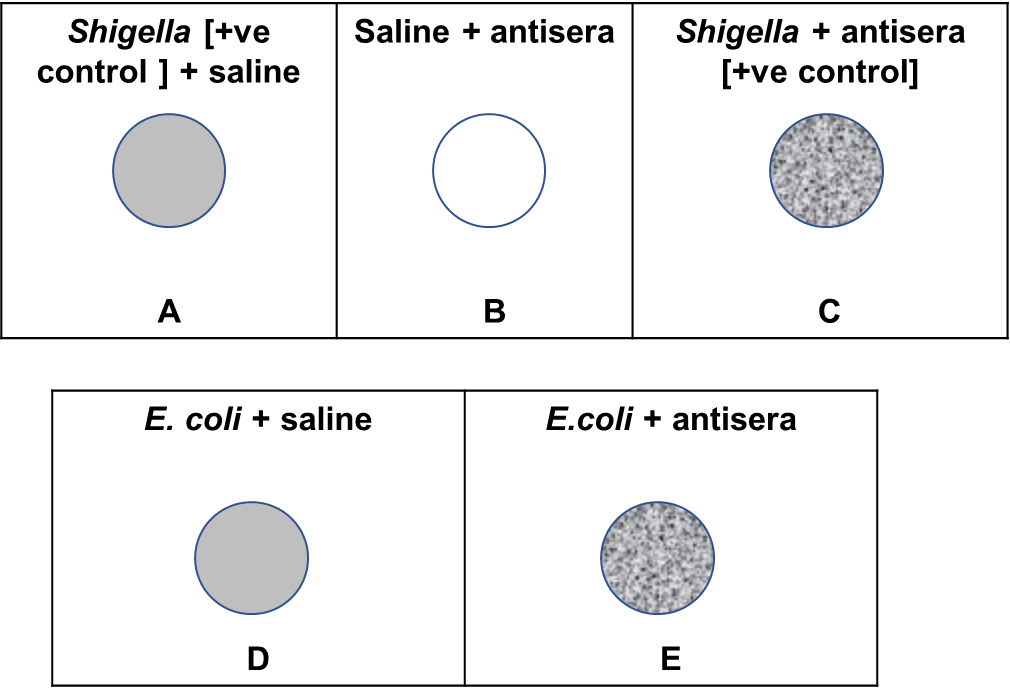
**

**Figure S3.** Controls to be tested for each serum.

**Table S1.** Expected results for QC organisms using polyvalent antisera.

| Type | Typing Sera | | | | | | | | |
| --- | --- | --- | --- | --- | --- | --- | --- | --- | --- |
|  | A | A1 | B | C | C1 | C2 | C3 | D |  |
| (-) Saline Control | - | - | - | - | - | - | - | - |  |
| *E. coli* ATCC 25922 | - | - | - | - | - | - | - | - |  |
| *S. dysenteriae* 4 CDC 402422 | + | ND | ND | ND | ND | ND | ND | ND |  |
| *S. dysenteriae* 8 ATCC 12021 | ND | + | ND | ND | ND | ND | ND | ND |  |
| *S. flexneri* 2B ATCC 12022 | ND | ND | + | ND | ND | ND | ND | ND |  |
| *S. boydii* 2 CDC 100705 | ND | ND | ND | + | ND | ND | ND | ND |  |
| *S. boydii* 9 ATCC 49812 | ND | ND | ND | ND | + | ND | ND | ND |  |
| *S. boydii* 15 ATCC  12034 | ND | ND | ND | ND | ND | + | ND | ND |  |
| *S. boydii* 17 ATCC 35965 | ND | ND | ND | ND | ND | ND | + | ND |  |
| *S. sonnei* CDC 100655 | ND | ND | ND | ND | ND | ND | ND | + |  |

  ND, Not Determined

**Table S2.** Expected results for QC organisms using monovalent antisera.

| Type | Typing sera | | | | | | Grouping sera | | |
| --- | --- | --- | --- | --- | --- | --- | --- | --- | --- |
|  | I | II | III | IV | V | VI | 3(4) | 6 | 7(8) |
| (-) Saline Control | - | - | - | - | - | - | - | - | - |
| *E. coli* ATCC 25922 | - | - | - | - | - | - | - | - | - |
| *S. flexneri* 1a CDC 08-2349 | + | ND | ND | ND | ND | ND | + | ND | ND |
| *S. flexneri* 2a CDC 06-3820 | ND | + | ND | ND | ND | ND | ND | ND | ND |
| *S. flexneri* 3a CDC 95-3254 | ND | ND | + | ND | ND | ND | ND | + | + |
| *S. flexneri* 4a CDC 07-4018 | ND | ND | ND | + | ND | ND | ND | ND | ND |
| *S. flexneri* 5a CDC  82-5099 | ND | ND | ND | ND | + | ND | ND | ND | ND |
| *S. flexneri* 6 CDC 95-3241 | ND | ND | ND | ND | ND | + | ND | ND | ND |

  ND, Not Determined

**Table S3.** Laboratory monitoring checklist.

| **Lab Monitoring** | | | | | | | | | |
| --- | --- | --- | --- | --- | --- | --- | --- | --- | --- |
| **Monitor**  **(Print Name)** |  | | **Site Name** | | | | | |  |
| **Date Lab Monitoring Visit Initiated** |  | | **Date Lab Monitoring Visit Complete** | | | | | |  |
| **Visit Time Point** |  | | **Corrective and Preventative Action Document required?** | | | | | | **□ Yes □ No** |
| **1. Safety** | | | | | | | | | |
|  | | **Yes** | | **No** | **N/A** | | **Comments** | | |
| a. Are technicians wearing suitable PPE for EFGH tasks? | |  | |  |  | |  | | |
| b. Tasks which include handling patient specimens are performed within a BSC? | |  | |  |  | |  | | |
| c. A sink and eye-wash station is readily available? | |  | |  |  | |  | | |
| d. Are cultures, stocks, and biohazardous waste decontaminated (chemically or autoclaving) prior to disposal? | |  | |  |  | |  | | |
| e. Are all sharps properly disposed into a sharp’s container? | |  | |  |  | |  | | |
| f. Are EFGH materials and samples properly labeled? | |  | |  |  | |  | | |
| **2. Personnel** | | | | | | | | | |
|  | | **Yes** | | **No** | | **N/A** | | **Comments** | |
| a. Is the number of available trained technicians sufficient to support the number of samples processed? | |  | |  | |  | |  | |
| b. Have all trained technicians present at site visit read and completed their DocuSign assignment for all active documents? | |  | |  | |  | |  | |
| c. Have all technicians assigned to the EFGH completed initial training/regular proficiency testing? | |  | |  | |  | |  | |
| **3. Equipment** | | | | | | | | | |
|  | | **Yes** | | **No** | | **N/A** | | **Comments** | |
| a. Is temperature regularly monitored for  -80°C Freezer(s)? | |  | |  | |  | |  | |
| b. Is temperature regularly monitored for 37°C Incubator(s)? | |  | |  | |  | |  | |
| c. Is equipment temperature regularly monitored for 2-8°C refrigerator(s)? | |  | |  | |  | |  | |
| d. Are all BSC calibrations in spec at time of visit? (Record expiry in comments) | |  | |  | |  | |  | |
| e. Are all pipettors in use in spec at time of visit? | |  | |  | |  | |  | |
| f. Has preventative maintenance (PM) occurred on all BSCs in the past 6 months - 1 year? | |  | |  | |  | |  | |
| g. Has PM occurred on all refrigerators in the past 6 months- 1 year? | |  | |  | |  | |  | |
| h. Has PM occurred on all freezers in the past 6 months- 1 year? | |  | |  | |  | |  | |
| i. Has PM occurred on all incubators in the past 6 months- 1 year? | |  | |  | |  | |  | |
| **4. Materials and Inventory** | | | | | | | | | |
|  | | **Yes** | | **No** | | **N/A** | | **Comments** | |
| a. Are all temperature sensitive materials stored at proper temperatures? | |  | |  | |  | |  | |
| b. Suitable storage space for all needed materials? | |  | |  | |  | |  | |
| c. Were the materials used in reviewed documentation past expiry? | |  | |  | |  | |  | |

| **5. Laboratory Documentation** | | | | |
| --- | --- | --- | --- | --- |
|  | Yes | No | N/A | Comments |
| - a. Printed Laboratory MOP available for all staff to view within lab? |  |  |  |  |
| b. All paper records used for patient testing reviewed and stored in an organized centralized location? |  |  |  |  |
| - c. All paper records used for technician training reviewed and stored in an organized and centralized location? |  |  |  |  |
| - d. All paper records used for proficiency testing reviewed and stored in an organized and centralized location? |  |  |  |  |
| **5a. Sample Processing Documentation**  **Reviewed worksheets are completed properly and have been reviewed and match** the completed CRF? Record number of documents reviewed in comments section (~10% of data) | | | | |
|  | Yes | No | N/A | Comments |
| - a. Accessioning Worksheet |  |  |  |  |
| - b. Stool Culture Worksheet |  |  |  |  |
| - c. AST Testing Worksheet |  |  |  |  |
| - d. Serology Testing Worksheet |  |  |  |  |
| **5b. QC and Test Preparation Documentation**  **Reviewed worksheets are completed properly and have been reviewed? (Record number of documents reviewed in comments section)** | | | | |
|  | Yes | No | N/A | Comments |
| - a. Media Preparation Log |  |  |  |  |
| - b. Temperature Sensitive Materials Log |  |  |  |  |
| - c. Serology QC Worksheet |  |  |  |  |
| - d. Shipping Manifest Form |  |  |  |  |
| - e. MacFarland Form |  |  |  |  |
| **6. Laboratory Flow in Lab Observation** | | | | |
|  | **Yes** | **No** | **N/A** | **Comments** |
| - a. Specimen transport temperature in range? |  |  |  |  |
| - b. Specimen transport time in range? |  |  |  |  |
| - c. Specimen packaged sealed upon receipt? |  |  |  |  |
| - d. Lab staff properly received sample organism? |  |  |  |  |
| - e. Was specimen accessioning completed per SOP? |  |  |  |  |
| - f. Was Stool Culture Workflow completed per SOP? |  |  |  |  |
| - g. Was MAC/XLD inoculation and interpretation completed per SOP? |  |  |  |  |
| - h. Was TSI inoculation and interpretation completed per SOP? |  |  |  |  |
| - i. Was urease Testing completed per SOP? |  |  |  |  |
| - j. Was MIO/MIU completed per SOP? |  |  |  |  |
| - k. Was LDC testing completed per SOP? |  |  |  |  |
| - l. Was oxidase testing completed per SOP? |  |  |  |  |
| - m. Was Single Biochemicals/ API completed per manufacturer? |  |  |  |  |
| - n. Real time Data Recording? |  |  |  |  |
| - o. Does data recorded reflect observed process? |  |  |  |  |
| - p. Sufficient space and equipment available to perform stool culture workflow? |  |  |  |  |
| Additional Comments  **□ N/A** | | | | |
